# Brain Responses Difference between Sexes for Strong Desire to Void: A Functional Magnetic Resonance Imaging Study in Adults Based on Graph Theory

**DOI:** 10.3390/jcm13154284

**Published:** 2024-07-23

**Authors:** Xiaoqian Ying, Yi Gao, Limin Liao

**Affiliations:** 1 Beijing Tsinghua Changgung Hospital, School of Clinical Medicine, Tsinghua University, Beijing 102218, China; yxqa03605@btch.edu.cn; 2 Rehabilitation School, Capital Medical University, Beijing Boai Hospital, China Rehabilitation Research Center, Beijing 100068, China; 3 Department of Neurourology, Beijing Boai Hospital, China Rehabilitation Research Center, Beijing 100068, China; 4 Department of Urology, Capital Medical University, Beijing 100068, China

**Keywords:** resting-state functional magnetic resonance imaging, brain–bladder control, graph theory, topologic properties, small-world network, desire to void

## Abstract

**Background:** The alternations of brain responses to a strong desire to void were unclear, and the gender differences under the strong desire to void remain controversial. The present study aims to identify the functional brain network’s topologic property changes evoked by a strong desire to void in healthy male and female adults with synchronous urodynamics using a graph theory analysis. **Methods:** The bladders of eleven healthy males and eleven females were filled via a catheter using a specific infusion and withdrawal pattern. A resting-state functional magnetic resonance imaging (fMRI) was performed on the enrolled subjects, scanning under both the empty bladder and strong desire to void states. An automated anatomical labeling (AAL) atlas was used to identify the ninety cortical and subcortical regions. Pearson’s correlation calculations were performed to establish a brain connection matrix. A paired *t*-test (*p* < 0.05) and Bonferroni correction were applied to identify the significant statistical differences in topological properties between the two states, including small-world network property parameters [gamma (γ) and lambda (λ)], characteristic path length (L_p_), clustering coefficient (C_p_), global efficiency (E_glob_), local efficiency (E_loc_), and regional nodal efficiency (E_nodal_). **Results:** The final data suggested that females and males had different brain response patterns to a strong desire to void, compared with an empty bladder state. **Conclusions:** More brain regions involving emotion, cognition, and social work were active in females, and males might obtain a better urinary continence via a compensatory mechanism.

## 1. Introduction

There are two primary functions for the lower urinary tract, named storage and voiding. Human beings can shift back and forth between the two phases, depend on the coordination of the brain, spinal cord pathway, peripheral nerve, bladder, urinary sphincter, and pelvic floor muscles. Significantly, any interruptions in functional coordination could lead to lower urinary tract dysfunctions (LUTDs), manifesting symptoms such as urinary frequency, urgency, incontinence, dysuria, and so on [1]. Bladder sensation is described in three patterns and indicated on the urodynamic graph: first sensation of filling, first desire to void, and strong desire to void [2,3]. Strong desire to void is introduced as a strong but not uncomfortable sensation to void at the ending of urinary bladder filling, which plays important roles in triggering a voiding phase. As reported in a study by Kaynar et al. [4], the highest maximum flow rate and average flow rate are obtained with a strong desire to void, suggesting its influence on the urinary voiding. Additionally, a strong desire to void is tightly related to urinary continence [5]. It was reported to impact the gait of individuals, with the sensory derived from multiple sclerosis and urinary disorders [6,7]. Urodynamic tests [8,9,10] were considered as an ideal method to estimate both storage and voiding function with a specific transurethral and a transrectal catheter. We employed the urodynamic test, a well-developed and applied urological clinical profile, to assess the function of the LUT.

In recent decades, a functional magnetic resonance imaging (fMRI) based on blood oxygen level-dependent (BOLD) is being applied to investigate the supraspinal control of the lower urinary tract (LUT) in healthy subjects and patients with LUTDs. Likewise, activations in the dorsal pontine, insula, prefrontal cortex, supplementary motor area, and the anterior and middle cingulate gyrus have been found in healthy subjects under a strong desire to void [11]. Abnormally activated brain regions and the alternations of functional connections in diseases with LUTD have been reported, including overactive bladder [12,13], stroke, and multiple sclerosis [14]. Nonetheless, the topologic property changes are barely known and sex differences in lower urinary tract regulation are also controversial. Although different patterns (especially the perfusion speed during the urodynamic study) of urinary bladder filling are generally considered as the potential influencing factors for urodynamic results, the alternations of brain responses resulting from these patterns are unclear as yet. A deeper understanding of the brain response to a strong desire to void may provide greater management strategies for LUT disorders.

A series of baseline brain fMRI studies concerning a strong desire to void are launched in healthy subjects to determine the central mechanism of healthy patients and patients with various LUTDs in the future. In the series, various patterns of urinary bladder filling are involved. In this article, with a repetitive infusion and withdrawal pattern, the functional brain network’s topologic property alternations evoked by a strong desire to void in healthy male and female adults will be reported.

## 2. Materials and Methods

### 2.1. Participants

All healthy participants were asked to fill a 3-day voiding diary. Notably, the physical tests were satisfactory, and no abnormal medical history existed. The 24-hour urine volume ranged between 1500 and 3000 mL Urination frequency was <eight times/day and ≤one time/night, with an average urine volume of >200 mL/time and no urine leakage. The exclusion criteria included having the following: (1) LUT symptoms; (2) neurological diseases or lesion, such as myelitis; (3) multiple sclerosis; (4) traumatic spinal cord injury; (5) tumors, especially pelvic tumors; (6) currently undergoing radiotherapy or chemotherapy; (7) taking medications or another substance which could impact the nervous system or urinary system; (8) pregnancy; (9) claustrophobia; and (10) having a medical aversion to scanning. Twenty-two healthy participants (11 males and 11 females) were enrolled. These participants signed an informed consent form, and the study was executed in accordance with the revised Helsinki Declaration, and the study’s ethics approval was provided by the Ethics committee of China Rehabilitation Research Center (IRB: 2017-002-1).

### 2.2. Image Acquisition and Preprocessing

All images were acquired on an Ingenia 3.0 Tesla scanner (Philips, Eindhoven, The Netherlands). Before the fMRI scans, the subjects were asked to void. Subjects lay supine while wearing headphones. A fixed device was used to limit the motion of the patient’s head. A 7 Fr double-lumen bladder catheter and a rectal catheter were inserted to monitor vesical pressure and intra-abdominal pressure with a portable urodynamic device (Laborie Medical Technologies, Williston, VT, USA). The procedure’s complete details were explained to the subjects, including closing their eyes and lying awake during scanning without any systematic thoughts. Structural T1 images (3D; repetition time = 7.8 ms; echo time = 3.8 ms; and flip angle = 8 ° were collected 10 min after voiding. Then, a gradient-echo, echo-plane imaging sequence (echo time = 30 ms; repetition time = 2000 ms; flip angle = 90 °; voxel size = 3 × 3 × 3.5 mm), was applied to all volunteers’ first resting-state fMRI scans.

**Figure 1:**
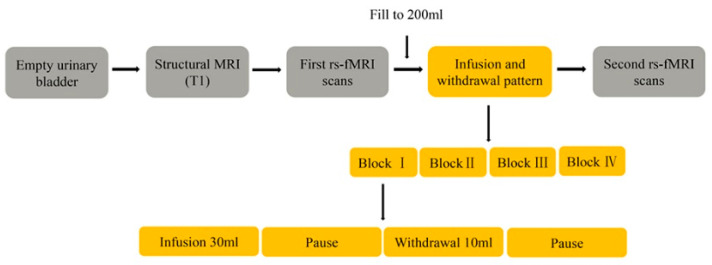
Bladder filling and fMRI protocol.

The scanning lasted 6 min 14 s, and a synchronous urodynamics evaluation was performed during the first resting-state functional brain scanning. After 2000 mL of 0.9%, sterile saline solution was infused into the subjects’ bladder by a syringe; the urinary bladder was filled via four blocks (Figure 1) with sterile saline solution. The second resting-state fMRI scans were performed using prior resting-state parameters, and a strong desire to void was reported. The urge to void was evaluated using a visual analog scale (score 0: empty bladder; score 6: strong desire to void; score 8: urge desire to void; score 10: pain), which had been applied in previous research [15,16]. Finally, bladder volume was measured after a voluntary elimination into a counting cup.

A standard preprocessing of acquisitions was performed with Statistical Parametric Mapping (version SPM8, https://www.fil.ion.ucl.ac.uk/spm/software/spm8, accessed on 30 September 2023). The first ten volumes were excluded from functional scans for magnetization equilibration. Slice timing correction, motion correction, the co-register between functional and structural scans, segmentation of structural images, normalization to Montreal Neurological Institute space, resampling with 3 mm × 3 mm × 3 mm voxel size, spatial smoothing with a 6 mm Gaussian kernel, detrending, and filtering with low-pass frequency filter (0.01–0.1Hz) were performed step by step. The data with head horizontal displacement in three-dimensional space > 2 mm and head rotation > 2° would be excluded during motion correction. Finally, nuisance signals resulting from head motion, white matter, cerebrospinal, and the global signal were regressed out.

### 2.3. Establishment of Connection Matrix/Networks

**Figure 2:**
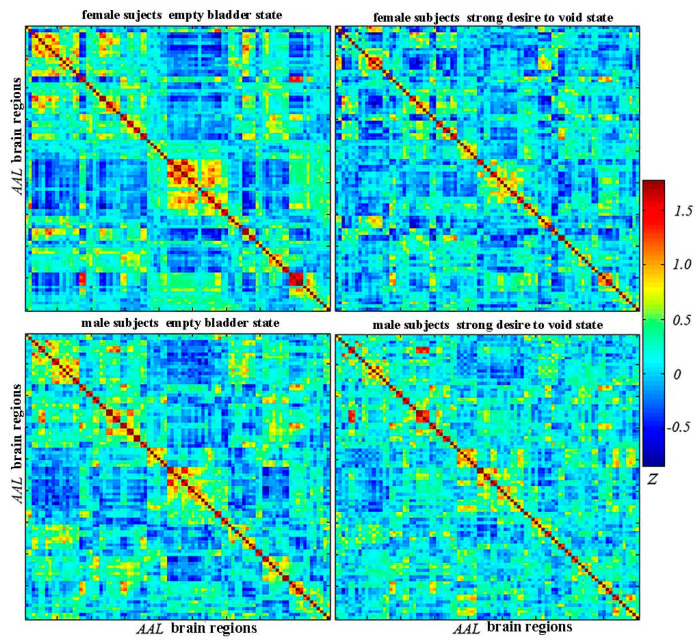
Brain functional connection matrix of healthy female and male subjects under both of the states.

The functional brain networks were established via graph theory methods, in which nodes represent various brain regions, and edges were modeled as a pairwise connection between nodes. The graph theory analysis was performed using a GRETNA 2.0.0 toolbox (https://www.nitrc.org/projects/gretna/, accessed on 3 October 2023). Regions of interest (ROIs), i.e., brain nodes, were initially identified using the automated anatomical labeling (AAL) atlas. The brain was divided into ninety anatomic regions (regions 1–90) using the AAL atlas. Then, the time series of the corresponding brain regions were extracted from the preprocessed data. Pearson’s correlation calculations were performed to determine the associations between achieving the time series of pairwise different brain regions. Furthermore, a 90 × 90 brain connection matrix (Figure 2) was achieved from each subject’s data. For normalization, a z-score was calculated with the Fisher r-to-z method. The graph analysis irrelevant or weak functional connections were excluded, with a proportional network threshold (sparsity T, 0.05–0.5; intervals, 0.05). When the Pearson’s correlation coefficient of individual pairs of connections was not greater than T, the corresponding connections were identified and removed. Otherwise, the functional connections were considered to exist. The threshold methods have been reported in the previous study [17].

### 2.4. Graph Analysis—Network Property Analysis

The twenty-two subjects were divided into the healthy male group and the healthy female group. Small-world properties, global efficiency (E_glob_), and local efficiency (E_loc_) were analyzed for the operational data after voiding and the acquired functional scans when subjects strongly desired voiding. The characteristic path length (L_p_) and the clustering coefficient (C_p_) are significant global metrics. L_p_, a parameter estimating the network’s integration function, is defined as the average shortest path lengths for all possible pairs of nodes. C_p_ expresses the density of connection of a node’s connection, which measures the network’s segregation. The more outstanding the C_p_ is, the denser the connections are. High E_glob_ and E_loc_ in the small-world network ensure the optimal function in segregation and integration. The C_p_ and L_p_ of the entire functional brain networks were normalized via being matched with randomly generated networks (n = 100) for the quantity of the small-world characteristics.

The gamma (γ) and lambda (λ) were obtained from the L_p_ and C_p_ of the entire network and random networks (γ = C_p real_/C_prand_; λ = L_p real_/L_p rand_). Sigma (σ, σ = γ/λ) was used to describe the small-world coefficient. If γ > 1, λ ≈1, and σ >1, the network has small-world properties. E_glob_ was used to estimate the network’s communication efficiency, and E_loc_ expressed the efficiency of the local subgraph of specific nodes that contains only the direct neighboring nodes. In terms of regional network properties, nodal efficiency (E_nodal_), evaluating a given node’s capacity for information communication with the other nodes, was calculated.

### 2.5. Statistical Analyses

For the statistical analysis, a paired *t*-test (*p* < 0.05) was performed for subjects’ characteristic data. And the Bonferroni correction between the empty bladder and strong desire to void was applied in both groups for the number of *t*-tests conducted due to the number of brain regions. The small-world topologic property parameters (γ and σ), L_p_, C_p_, E_glob_, E_loc_, and regional E_nodal_ involved.

## 3. Results

The resting state fMRI data of eleven males and eleven females were analyzed in the study. A male and a female were excluded for head motion (head displacement > 2 mm and head rotation > 2°). Ten females (mean age: 51.4 years, age range: 35–65 years) and ten males (mean age: 51.6 years, age range: 45–64 years) were available for final statistical analysis (Table 1). There was no statistical difference between both groups in the age (*p* = 0.09), the sensory score of the visual analog scale (*p* = 0.23), and the bladder volume under a strong desire to void (*p* = 0.19). The urodynamic findings showed the stationary and normal urinary storage in all healthy subjects, without detrusor overactivity detected.

### 3.1. Global Functional Network Properties

**Figure 3:**
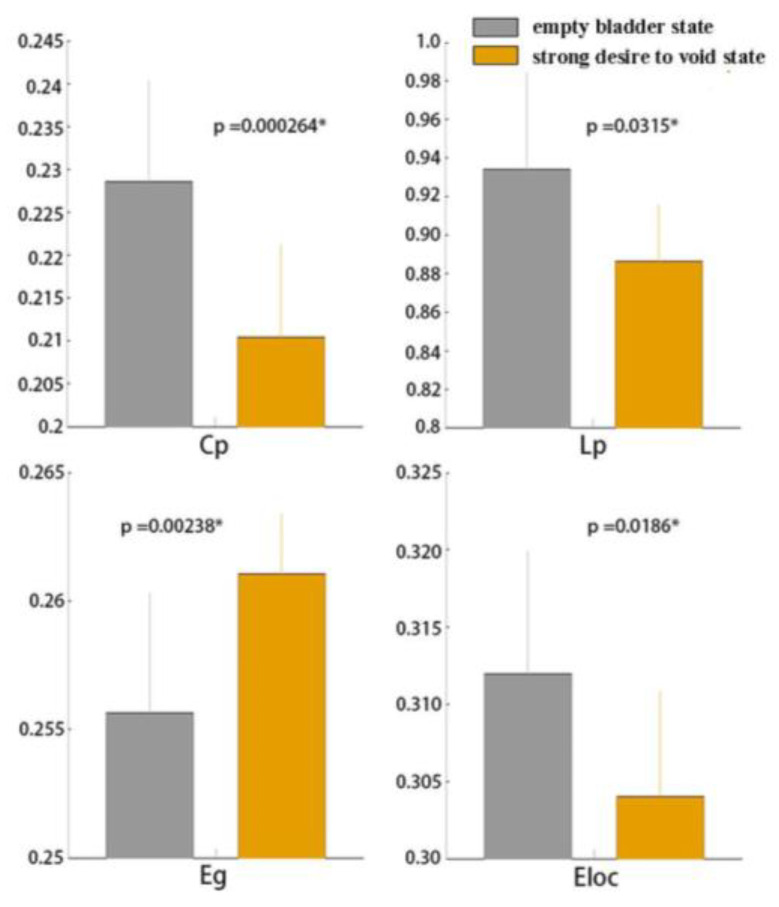
The C_p_, L_p_, E_g_, and E_loc_ in healthy female subjects between both of the states. Legend: It showed that the significantly increased C_p_, L_p_, E_glob_ and decreased E_loc_ in these females. C_p_, clustering coefficient; L_p_, characteristic path length; E_glob_, global efficiency; E_loc_, local efficiency (*p* < 0.05) (y axis label is the σ value; * represents statistically significant data).

**Figure 4:**
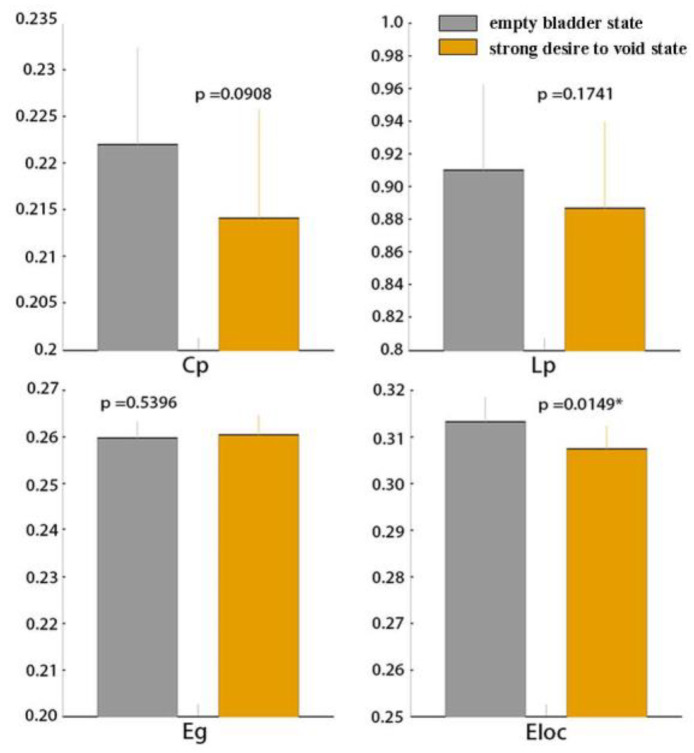
The C_p_, L_p_, E_g_ and E_loc_ in healthy male subjects between both of the states. Legend: It showed that the significantly decreased C_p_ in the female subjects, without significant changes in C_p_ L_p_, E_glob_. C_p_, clustering coefficient; L_p_, characteristic path length; E_g_, global efficiency; E_loc_, local efficiency (*p* < 0.05) (y axis label is the σ value; * represents statistically significant data).

After normalizing with random networks, higher C_p_ (γ > 1) and L _p_ (λ ≈ 1) for network sparsity was observed in all subjects under the empty bladder state (mean ± SD, γ in female group: 1.48 ± 1.95; γ in the male group: 2.00 ± 2.25; λ in the female group: 0.30 ± 1.14; λ in the male group: 0.31 ± 1.14) and the strong desire to void state (mean ± SD, γ in the female group: 1.52 ± 2.01; γ in male group: 1.55 ± 2.03; λ in female group: 0.23 ± 1.10; λ in male group: 0.22 ± 1.10). Small-world properties (σ >1) were detected in both groups under the empty bladder state (mean ± SD, σ in the female group: 0.63 ± 1.59; σ in the male group: 0.94 ± 1.81) and the strong desire to void state (mean ± SD, σ in the female group: 0.77 ± 1.70; σ in the male group: 0.86 ± 1.73). There were no significant differences in the small-world coefficient (σ, *p* > 0.05) between the states in the two groups. Compared with the empty bladder state, significantly decreased C_p_, L_p_, E_loc_ and increased E_glob_ (*p* < 0.05; *p* in C_p_: 0.00, *p* in Lp: 0.03, *p* in E_loc_: 0.02, *p* in E_glob_: 0.00) were detected in the female group under a strong desire to void (Figure 3). In the male group, significantly decreased E_loc_ was detected in the strong desire to void state (*p* = 0.02). There were no statistical differences between the two states in C_p_, Lp, and E_glob_. (*p* > 0.05; *p* in C_p_: 0.09, *p* in Lp: 0.17, *p* in E_glob_: 0.54) (Figure 4).

### 3.2. Regional Nodal Efficiency of Functional Brain Networks

**Figure 5:**
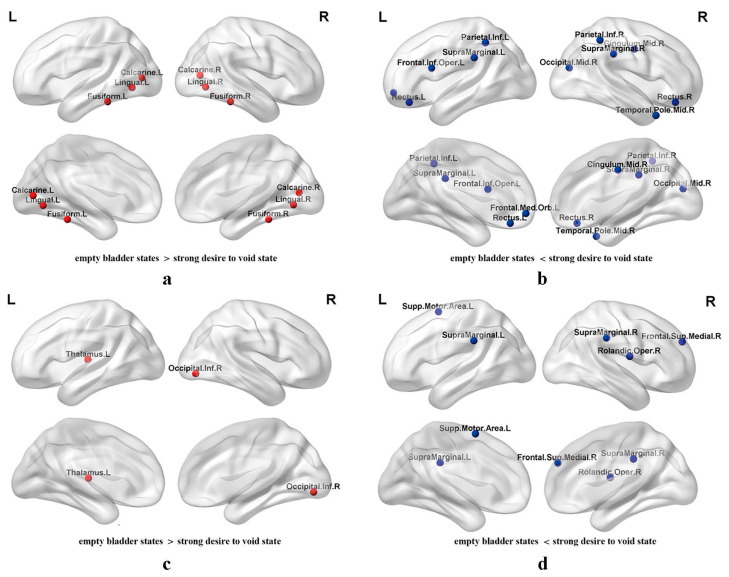
The brain regions with an alternated E_nodal_ in the healthy male subjects between both of the states. Legend: (**a**) For female subjects, a larger E_nodal_ in bilateral calcarine fissure and surrounding cortex, lingual gyrus, and fusiform gyrus were detected under the empty bladder state. (**b**) Under the strong desire to void state, a larger E_nodal_ in the left inferior frontal gyrus and the orbital part of middle frontal gyrus, right median cingulate, middle occipital gyrus and middle temporal gyrus, and bilateral gyrus rectus, inferior parietal gyrus and supramarginal gyrus was detected in healthy females. (**c**) The larger E_nodal_ in the right inferior occipital gyrus and thalamus of male subjects under the empty bladder state. (**d**) The significantly increased E_nodal_ of healthy male subjects presented in right frontal operculum and medial superior frontal gyrus, left supplementary motor area and the bilateral supramarginal gyrus (*p* < 0.05).

In the female group, the significant increase in E_nodal_ (*p* < 0.05 after FDR correction) under the strong desire to void state was observed in the following brain regions compared to the empty bladder: (1) left prefrontal gyrus (Frontal_Inf_Oper_L and Frontal_Med_Orb_L); (2) bilateral gyrus rectus (Rectus_L and Rectus_R); (3) median cingulate and paracingulate gyrus (Cingulum_Mid_R); (4) middle occipital gyrus (Occipital_Mid_R); (5) bilateral inferior parietal gyrus (Parietal_Inf_L and Parietal_Inf_R); (6) bilateral supramarginal gyrus (SupraMarginal_L and SupraMarginal_R); and (7) middle temporal gyrus (Temporal_Pole_Mid_R). Furthermore, a significant decrease in E_nodal_ was detected in the bilateral calcarine fissure and surrounding cortex (Calcarine_L and Calcarine_R), lingual gyrus (Lingual_ L and Lingual_R), and fusiform gyrus (Fusiform_L and Fusiform_R) (Figure 5a,b).

Notably, for the male group, a significant increase in E_nodal_ (*p* < 0.05 after FDR correction) in the strong desire to void state was detected in the right frontal operculum (Rolandic_Oper_R); left supplementary motor area (Supp_Motor_Area_L); medial superior frontal gyrus (Frontal_Sup_Medial_R); and the bilateral supramarginal gyrus (SupraMarginal_L and SupraMarginal_R). However, a decrease in E_nodal_ was observed in the right inferior occipital gyrus (Occipital_Inf_R) and left thalamus (Thalamus_L) (Figure 5c,d).

## 4. Discussion

Although more than twenty studies addressed brain fMRI findings on urinary bladder control in the past decades, simultaneous healthy females and males are barely enrolled and reported [18]. Because bladder control involves physiological behavior and psychosocial factors, the healthy female and male subjects were analyzed under the same scanning parameters and their different brain response patterns were described without comparison between the two groups. The differences in the brain topologic property alternations evoked by the strong desire to void state may provide an understanding for the central-LUT control fundamental mechanism in healthy women and men.

### 4.1. Global Graph Metrics

In this study, the repetitive infusion and withdrawal pattern was used to activate the regions related to LUT control. Small-world network properties were observed in the empty bladder and strong desire to void states in both groups. High C_p_ and low L_p_ are outstanding characteristics of the small-world structure, which are optimized for information processing. Balanced functional integration and segregation are observed in the small-world networks according to previous general assumptions. High E_glob_ and E_loc_ are detected in the small-world networks, which demonstrate higher efficiency in global and local information communication than the regular network (with low E_glob_ and high E_loc_) and the random network (with high E_glob_ and low E_loc_).

Although the small-world features were detected in both states, the significantly decreased C_p_ and E_loc_ were observed in the females provoked by a strong desire to void compared with the empty bladder state, which revealed the lower capacity in local information processing and the decreased efficiency in local information transmission. The significantly decreased L_p_ and increased E_glob_ in globally connected graphs suggested the higher capacity in the information processing and higher efficiency in global information communication transmission and a better functional integration in the female group.

In the male group, the decreased E_loc_ was observed under the strong desire to void state compared with the empty bladder state, revealing the lower efficiency in local information transmission. These results implied the decreased trend in functional segregation in the male group.

### 4.2. Regional Nodal Metrics

In the two groups, the significantly increased E_nodal_ under the strong desire to void state was detected in the bilateral frontal areas (left inferior frontal gyrus, orbital part of the middle frontal gyrus, right median cingulate gyrus, and bilateral gyrus rectus in females; and right frontal operculum, medial superior frontal gyrus, and left supplementary motor area in males) and the bilateral supramarginal gyrus. Meanwhile, the middle occipital gyrus, middle temporal gyrus, and bilateral inferior parietal gyrus presented increased E_nodal_ in the female subjects. And, in the male group, the increased E_nodal_ presented in the bilateral supramarginal gyrus. The significantly decreased E_nodal_ in the female group was detected in the bilateral occipital lobe (calcarine fissure and surrounding cortex and lingual gyrus involved), and fusiform gyrus. The decreased E_nodal_ in the male group presented in the right occipital lobe (inferior occipital) gyrus and thalamus.

Present clinical human trials and animal experiments [19,20,21] have shown that there is a notable voiding reflex between the bladder and the midbrain periaqueductal gray (PAG). During the bladder storage, the afferent filling sensory signals caused bladder distension until the volume threshold in PAG is exceeded. The voiding reflex is provoked to relax the urethral sphincter and contract bladder detrusor, and voiding begins. Then, urinary bladder storage restarts when it is empty. In fact, the higher central mechanism works in the entire bladder filling and voiding process.

The prefrontal cortex (PFC) is crucial for the LUT control. The PFC is involved in human personality, decision-making, and social behavior. Significantly, the cortex has been presumed to control voluntary action, including deciding to void [22]. In previous research [23], as the urinary bladders were passively infused, heathy female brain responses to larger bladder volume increased in the orbitofrontal cortex. But, in patients with bladder overactivity and poor bladder control have weaker responses in the region. The lateral PFC concerns cognition, especially in work memory [24]. The region has been proven to have comprehensive connections with the limbic system, and these diverse connections are possibly relevant to various motor and reward functions [25]. Neuroanatomical links between PFC and PAG are suspected to be related to suppressing voiding, which suggests the important role of the PFC in urinary continence [26].

Positron-emission tomography (PET) and single photon emission computed tomography (SPECT) studies report the activation of the bilateral inferior frontal gyrus, but the evidence is absent in the male fMRI [11], and the increased E_nodal_ only presents in the female subjects in our study. Yin et al. demonstrate that the middle temporal gyrus and the right inferior frontal gyrus inhibit detrusor contraction together during urinary bladder storage in healthy subjects [27]. Patients with detrusor overactivity are found weaker in the two regions [28]. Medial PFC was an essential part of the default mode network (DMN) [29]. Interoceptive and spatial representations of the body were integrated into the DNM, including the bladder sensory [30]. When it came to self-awareness and self-reflection under a resting state, the DNM is activated. Additionally, the region works in the cognitive process, regulating emotion and sociability [31]. Medial prefrontal gyrus lesions are found to result in short-term incontinence in adults. Nevertheless, the white-matter lesion in the medial PFC also leads to long-term urinary bladder dysfunction [32]. And fMRI research shows that the region is deactivated under the full urinary bladder in patients with urgency incontinence [33]. Visceral perception, including the urinary bladder filling sensory, can be integrated with exteroceptive and interoceptive signals [16]. The frontal operculum is a region adjacent to the insula, which involves the awareness and processing of interoceptive signals [34].

The gyrus rectus, as a subregion of orbitofrontal cortex, mediates the interaction between the visceromotor centers and the prefrontal sensory signals via the hypothalamus’s descending pathway and the brainstem [35]. The ventromedial PFC is proven to connect with the limbic system and other brain regions, which determine its vital roles in LUT control. Additionally, the gyrus rectus is reckoned to predict adult difficulties in social functioning and reduced cognitive empathy [36]. The abnormalities in gyrus rectus, as well as orbitofrontal and cingulate gyrus, are associated with depression [37].

The cingulate gyrus as a component of the limbic system is known for multiple functions, such as the mediation of emotional and autonomic responses to external stimuli and processing the information from the bladder to maintain urinary continence and impact the urge to void [38,39]. The cingulate gyrus is involved in visceral stimulation, and the urge to void was considered a nonpainful visceral stimulation [12]. As mentioned, the anterior or median cingulate cortex controls the heart rate via sympathetic mechanisms [40,41]. The dorsal anterior or median cingulate cortex is speculated to control the lower urinary tract via the same mechanism, which cannot be precisely identified now. Under a strong desire to void or urgency, the activated dorsal anterior cingulate cortex would facilitate urinary continence by urinary sphincter contraction and bladder relaxation [42]. Moreover, Cohen and co. [43] suggest that the cingulate gyrus and premotor cortex play important roles in regulating selective attention under task–conflict conditions. During the process for the study, the subjects who had the strong desire to void knew voiding in the scanner was inappropriate. In our study, we detected an increased E_nodal_ in the right median cingulate gyrus in the female group. So, with all that, more frontal regions changed with the strong desire to void in female subjects, particularly in areas involving emotion, cognition, and social function.

The pelvic floor muscles, which are important in stress urinary incontinence, cannot be isolated. The supplementary motor area (SMA), a location adjacent to the dorsal anterior cingulate cortex, showed the increased E_nodal_ under the strong desire to void in the male subjects; this was absent in the female pattern. The SMA showed the activated response during the pelvic floor muscle’s voluntary contraction [28,34]. Gehring and Knight [44] have suggested the activation in the premotor cortex worked with the cingulate gyrus to monitor behavior and guiding compensatory system; the gender difference may partly result from the compensatory system. Under a strong desire to void, the subjects’ brains monitored and regarded that voiding in the scanner was inappropriate, the pelvic floor tissues contraction might be initiated as a compensatory mechanism to resist urine leakage. Moreover, the activated cingulate gyrus and SMA contribute to the contraction of the urethral sphincter and detrusor suppression [45].

The supramarginal gyrus, located in the inferior parietal lobe (IPL), shows a vital association with proprioception. A recent study has suggested that the regions were activated under the condition of visceral perception, such as heartbeat [46]. The multifunction of IPL has been revealed in multisensory integration, spatial attention, higher cognitive functions, and oculomotor control [47,48,49]. The PET and fMRI studies show that the IPL also responded to urinary bladder cooling during the bladder storage using ice water [15,50]. Meanwhile, the human fusiform gyrus is a region concerning object recognition and functional definition, which often changes under high spatial frequency stimuli and interacts with the occipital lobe on visual tasks [51,52]. The occipital cortex change has been mentioned in previous research, but the mechanism was not systematically discussed [53,54].

Distinctly, regional E_nodal_ in the male group’s thalamus was decreased under the strong desire to void state. No difference is observed between urinary bladder filling and emptying in the female group. As a relay station, the thalamus transmits the sensory signals from PAG to the insula, lateral PFC, and medial PFC in animals and human beings [11,55,56,57]. The decreased E_nodal_ in the thalamus suggests the lower efficiency in information transmission in regionally connected graphs. The study of Kuhtz-Buschbeck et al. [42] has similar gender differences, in which the thalamus in healthy males is less activated under the urge to void compared with healthy females.

### 4.3. Limitations

Despite the differences in anatomy, social psychology, and behavior, a consensus has not been reached about the gender differences in central LUT control. The presence of multifactorial differences makes the fMRI results of comparisons between the two groups more difficult to interpret. This is why we described the brain response to the strong desire. Nevertheless, more information might be lost due to the shortage of intergroup comparison. On the other hand, to ensure the lower urinary tract function during fMRI scanning, urodynamic tests were conducted whereby specialized tubes for pressure measurement were inserted into the bladder and rectum. Despite the same sensory score on the visual analog scale and bladder volume, diverse anatomy and unphysiological prefusion with saline might potentially affect the brain activities. Further studies are needed to verify their specific effects on brain function. Secondly, Blok and his coworkers [48,56] have indicated different levels of activation in the insula, hypothalamus, and PAG during micturition and urine withholding between healthy females and males in their PET study. A meta-analysis of neuroimaging studies has revealed that activated clusters in the brainstem (periaqueductal gray and rostral pons), thalamus, insula, and cerebellum respond to the urinary bladder filling, and no significant difference in brain activation between female and male subjects is detected [49]. Although only cortical and subcortical regions without the pons and cerebellum were involved in our study, its results have provided the evidence for the gender difference in responses to the strong desire to void under the pattern. Additionally, the age range of the subjects is rather limited and some subjects are not young. Although these subjects are healthy, old age is associated with urinary control problems, and they might be more susceptible and anxious. This aspect might impact underlying gender differences in brain activation. Finally, the sample size was small, which may be a potential limitation of the study. On the other hand, due to the AAL atlas we used to define the regions of interest, only brain activity in the cortical and subcortical regions are in focus. We will consider future studies covering the cerebellum and pons to obtain more integrated information.

## 5. Conclusions

With the repetitive infusion and withdrawal pattern, we detected different functional topologic property alternations in the healthy female and male subjects between the strong desire to void and empty bladder states in our study. More brain regions involving emotion, cognition, and social work were changed in females, and males might obtain a better urinary continence via a compensatory mechanism. The baseline findings in healthy females and males might help in understanding the underlying pathogenesis in LUTD patients during the urodynamic tests.

## Figures and Tables

**Table 1:** The characteristics of the healthy female and male subjects.

Characteristics	Females	Males
Numbers	10	10
Age (mean ± SD)	51.4 ± 5.9	51.6 ± 5.4
Education (years)	7.2 ± 1.6	10.9 ± 3.7
Mean score of visual analog scale	6.7 ± 0.9	6.9 ± 0.8
Bladder volume under strong desire to void (mL)	436.0 ± 129.7	407.0 ± 139.1
Adverse events after the study		
Frequency/Urgency/Leakage (times)	0	0
Dysuria/Urinary retention/Hematuria	0	0

## Data Availability

The data and materials on the work are available from the corresponding author for reasonable request.
